# Retraction: Adaptive double threshold energy detection based on Markov model for cognitive radio

**DOI:** 10.1371/journal.pone.0222624

**Published:** 2019-09-20

**Authors:** The PLOS ONE Editors

Following publication of this article [1], concerns were raised regarding substantial overlap in the equations and data with work reported in a previous publication by some of the same authors [2], which was not cited or discussed.

Following evaluation by a member of the Editorial Board, it was determined that the approach and majority of the analyses in the *PLOS ONE* article are reported in [2]. Additionally, it was noted that there is some text overlap in the “Markov model based on time-varying parameters for spectrum occupancies” section compared with a number of previously published works by other authors [3–5]; that a number of equations as well as Algorithm 1 are equivalent or similar to those reported in [3]; and that the unique contribution of this work [1] in the context of existing literature is not accurately and clearly identified.

In light of the concerns about substantial redundancy of this work, the *PLOS ONE* Editors retract the article.

The authors did not comment on the retraction decision or could not be reached.
